# Extensively Invasive Gallbladder Cancer from Intracholecystic Papillary Neoplasm Treated with Pylorus-Preserving Pancreaticoduodenectomy and Extended Cholecystectomy: A Case Report and Literature Review

**DOI:** 10.1155/2023/5825045

**Published:** 2023-06-24

**Authors:** Hideki Kumagai, Akira Umemura, Hiroyuki Nitta, Hirokatsu Katagiri, Masao Nishiya, Noriyuki Uesugi, Tamotsu Sugai, Akira Sasaki

**Affiliations:** ^1^Department of Surgery, School of Medicine, Iwate Medical University, 2-1-1 Idaidori, Yahaba, Iwate 028-3695, Japan; ^2^Department of Molecular Diagnostic Pathology, Iwate Medical University, 2-1-1 Idaidori, Yahaba, Iwate 028-3695, Japan

## Abstract

**Background:**

Intracholecystic papillary neoplasm (ICPN) is a rare tumor first classified by the World Health Organization in 2010. ICPN is a counterpart of the intraductal papillary mucinous neoplasm of the pancreas and intraductal papillary neoplasm of the bile duct. Previous reports on ICPN are limited; thus, the diagnosis, surgical intervention, and prognosis are controversial. Here, we report an extensively invasive gallbladder cancer arising in ICPN treated with pylorus-preserving pancreaticoduodenectomy (PPPD) and extended cholecystectomy. *Case Presentation*. A 75-year-old man presented to another hospital with jaundice for 1 month. Laboratory findings showed elevated total bilirubin, 10.6 mg/dL and carbohydrate antigen 19-9, 54.8 U/mL. Computed tomography showed a well-enhanced tumor located in the distal bile duct and dilated hepatic bile duct. The gallbladder wall was thickened and homogeneously enhanced. Endoscopic retrograde cholangiopancreatography revealed a filling defect in the distal common bile duct, and intraductal ultrasonography showed a papillary tumor in the common bile duct, indicating tumor invasion of the bile duct subserosa. Subsequent bile duct brush cytology revealed adenocarcinoma. The patient was referred to our hospital for surgical treatment and underwent an open PPPD. Intraoperative findings showed a thickened and indurated gallbladder wall, suggesting concurrent gallbladder cancer; thus, the patient subsequently underwent PPPD and extended cholecystectomy. Histopathological findings confirmed gallbladder carcinoma originating from ICPN, which extensively invaded the liver, common bile duct, and pancreas. The patient started adjuvant chemotherapy (tegafur/gimeracil/oteracil) 1 month after surgery and had no recurrence at follow-up after 1 year.

**Conclusions:**

Accurate preoperative diagnosis of ICPN, including the extent of tumor invasion is challenging. To ensure complete curability, the development of an optimal surgical strategy considering preoperative examinations and intraoperative findings is essential.

## 1. Introduction

Intracholecystic papillary neoplasm (ICPN) is a relatively new concept established by the 2010 World Health Organization (WHO) classification [1]. According to this classification, ICPN is recognized as a counterpart of intraductal papillary mucinous neoplasm in the pancreas and intraductal papillary neoplasm of the bile duct [1]. Tumors are considered premalignant lesions [2, 3].

ICPN is rare, accounting for 0.4–1.5% of cholecystectomies and 6.4% of gallbladder cancers [1–3]. Therefore, there are limited previous studies on the diagnosis or surgical management of ICPN, and the prognosis of ICPN remains controversial.

Here, we report a rare case of extensively invasive gallbladder cancer arising in ICPN treated with pylorus-preserving pancreaticoduodenectomy (PPPD) and extended cholecystectomy and review the previous literature.

## 2. Case Presentation

**Figure 1:**
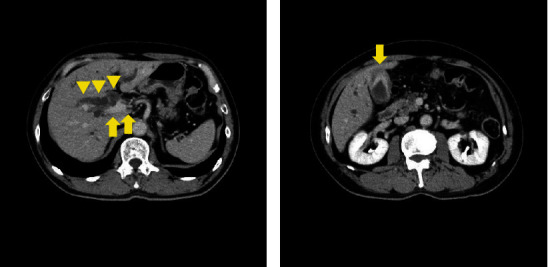
Enhanced CT findings. (a) A well-defined tumor from a cystic duct to a distal bile duct was observed (yellow arrow), and a hepatic-sided bile duct seen from the tumor was dilated. (b) Gallbladder mucosa was well-contrasted, and the wall was thickened (yellow arrow). CT: computed tomography.

**Figure 2:**
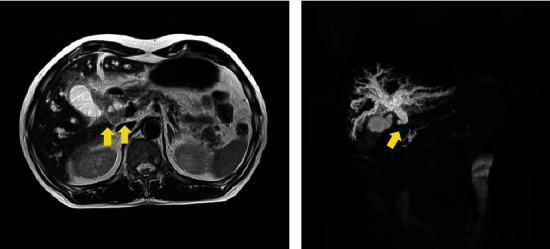
MRI and MRCP findings. (a) The tumor located in the distal bile duct had a low T2 signal (yellow arrow). (b) MRCP showed a filling defect of the distal bile duct and dilation of the hepatic-sided bile duct seen from the tumor (yellow arrow). MRCP: magnetic resonance cholangiopancreatography; MRI: magnetic resonance imaging.

**Figure 3:**
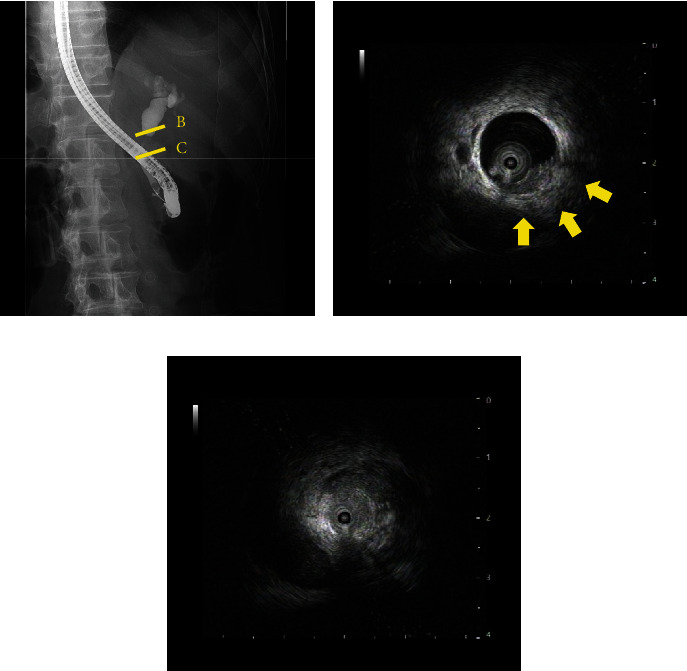
ERCP and IDUS findings. (a) ERCP showed a filling defect of the distal bile duct; the cystic duct was invisible. (b) IDUS showed that the tumor had invaded the bile duct subserosa (yellow arrow). (c) The patient's distal bile duct was almost obstructed by the tumor. ERCP: endoscopic retrograde cholangiopancreatography; IDUS: intraductal ultrasonography.

A 75-year-old man presented to the hospital with jaundice that had been present for 1 month. He had a medical history of hypertension and non-tuberculosis mycobacterial infection but no surgical history. Laboratory findings showed elevated levels of serum bilirubin and liver enzymes: total bilirubin, 10.6 mg/dL; aspartate aminotransferase, 68 IU/L; alanine aminotransferase, 119 IU/L; and gamma-glutamyl transpeptidase, 393 IU/L. Serum carbohydrate antigen 19-9 was also elevated (54.8 U/mL), though carcinoembryonic antigen was within the normal range. Enhanced computed tomography (CT) revealed a well-enhanced tumor in the distal bile duct and dilation of the hepatic bile duct (Figure 1(a)). In addition, the gallbladder mucosa was thickened, with a homogeneous contrast effect (Figure 1(b)). Magnetic resonance imaging (MRI) showed a low T2 signal tumor in the common bile duct (Figure 2(a)), and magnetic resonance cholangiopancreatography (MRCP) showed dilation of the hepatic-sided bile duct from the tumor (Figure 2(b)). The thickened gallbladder walls had a homogeneous low T2 signal; however, liver invasion by the tumor was not significant. Endoscopic retrograde cholangiopancreatography (ERCP) revealed a filling defect in the distal bile duct, and the cystic duct was invisible (Figure 3(a)). Moreover, intraductal ultrasonography (IDUS) showed a papillary tumor in the common bile duct and indicated invasion of the bile duct subserosa (Figures 3(b) and 3(c)). Subsequent bile duct brushing cytology on ERCP and IDUS revealed adenocarcinoma, and the patient was diagnosed with distal bile duct cancer associated with adenomyomatosis of the gallbladder.

**Figure 4:**
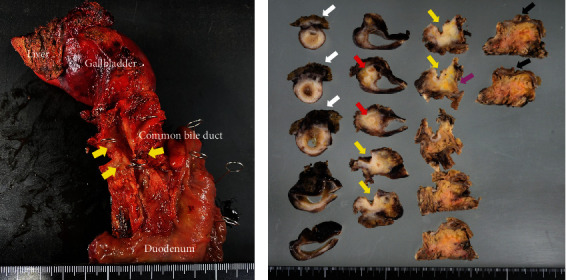
Macroscopic findings. (a) Macroscopic findings revealed a papillary tumor that had invaded the common bile duct. The distal bile duct lumen was severely constricted by the tumor invasion (yellow arrow). (b) Cross section of the resected specimen revealed that the white tumor extensively invaded the liver (white arrow), cystic duct (red arrow), common bile duct (yellow arrow), and pancreas (pink arrow). Black arrows point to the papilla of Vater.

**Figure 5:**
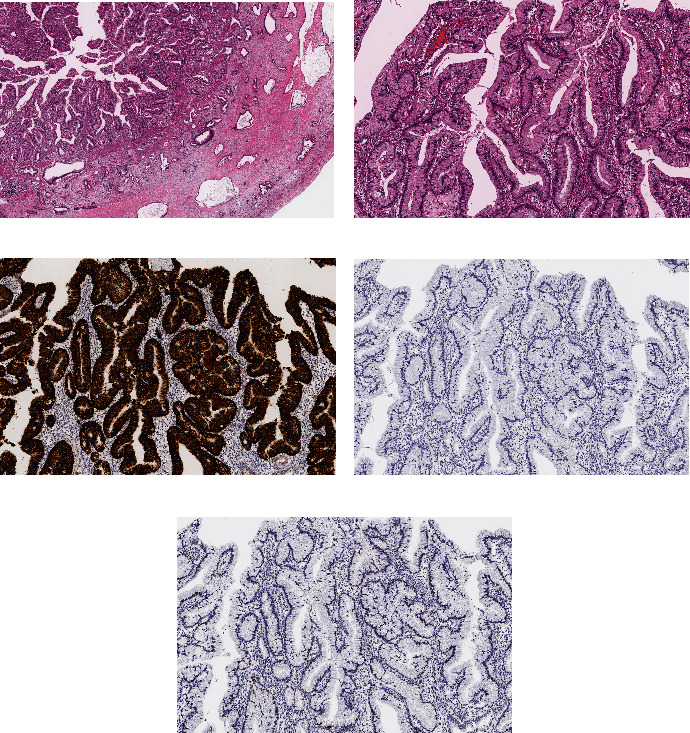
Histopathological images showed that the tumor was papillotubulary and had broad-based growth of columnar cells with mucus production in the gallbladder; hematoxylin and eosin staining: (a) 20× and (b) 100×. Immunohistochemical analysis showed the tumor cells were positive for mucin (MUC)5AC staining (c), but negative for MUC2 (d) and p53 (e) (all 100×).

The patient was referred to our hospital for surgical treatment, and we planned to perform PPPD for distal bile duct cancer with curative intent. Intraoperative findings revealed a thickened and indurated gallbladder wall, suggesting the coexistence of gallbladder cancer; thus, we performed PPPD and extended cholecystectomy. Intraoperative frozen-section analysis of the cut end of the hepatic bile duct was negative for the tumor. A macroscopic examination of the resected specimen revealed a papillary tumor that had extensively invaded the liver, cystic duct, bile duct, and pancreas (Figures 4(a) and 4(b)). Permanent histopathological findings indicated that the tumor was papillotubulary and had broad-based growth of columnar cells with mucus production in the gallbladder (Figures 5(a) and 5(b)). In addition, metastasis to the lymph nodes of the hepatoduodenal ligament was observed. Immunohistochemical analysis revealed that the tumor cells were positive for mucin (MUC)1, MUC5AC, and MUC6, but negative for MUC2 and p53 (Figures 5(c), 5(d), and 5(e)). Finally, the diagnosis of gastric-type ICPN was established according to the 2010 WHO classification. The postoperative course was uneventful, and the patient started adjuvant chemotherapy (tegafur/gimeracil/oteracil) 1 month after surgery. At the follow-up after 1 year, the patient had no recurrence.

## 3. Discussion

We report the case of a patient with extensively invasive gallbladder cancer originating in ICPN treated with PPPD and extended cholecystectomy. Adsay et al. reported that invasiveness was observed in 55% of ICPN cases [1]; however, most patients with ICPN are found at an early stage incidentally by imaging studies, as mentioned below, and reports on advanced cases are limited [4–7]. We searched for previous reports on ICPN in PubMed using the keywords “intracholecystic papillary neoplasm” or “intracystic papillary neoplasm” and reviewed 39 cases [4–39], including the present case diagnosed as ICPN histopathologically (Table 1). The mean age of patients with ICPN was 66.3 years, and female patients outnumbered male patients, as previously reported [3]. Approximately half of the ICPNs have invasive components, as found by Adsay et al., however, our patient was the only patient with lymph node metastasis in our review.

Moreover, Adsay et al. reported that approximately half of the ICPNs develop in the right upper abdominal region, and the other half are incidentally found by imaging studies [1], which is similar to our findings summarized in Table 1. Conversely, jaundice is an uncommon symptom in ICPN, and there are few previous reports in the literature [4, 5, 25, 27]. Of the four patients, two patients suffered from jaundice resulting from a protruding tumor from the gallbladder to the common bile duct [4, 5]. Interestingly, the other two developed jaundice due to mucus production from the tumor [25, 27]. In the present case, histopathological findings revealed that the tumor had extensively invaded the bile duct; thus, the patient had obstructive jaundice owing to tumor invasion of the common bile duct rather than a protruding tumor from the gallbladder. Protruding or advanced tumors associated with ICPN can cause obstructive jaundice; moreover, mucus production from ICPN can lead to jaundice.

Distinguishing between ICPN and other gallbladder tumors using imaging studies is difficult. In our review, only 15.8% of patients with ICPN were diagnosed accurately before surgery. According to previous reports, ICPN is well-defined on enhanced CT and presents high or low T2 signal intensity and high diffusion-weighted imaging signal intensity on MRI [4, 7]. Fluorodeoxyglucose (FDG) accumulation in ICPN has been observed on FDG-positron emission tomography [5, 28]. However, these are non-specific findings that can be observed in other gallbladder tumors. Moreover, histopathological examinations, including cytology and biopsy are not diagnostic in terms of distinguishing between ICPN and other types of gallbladder carcinomas [4–7]. However, endoscopic ultrasound (EUS), including IDUS or peroral cholangioscopy (POCS) has shown the presence of a papillary tumor in most patients diagnosed with ICPN preoperatively [4–6, 25, 27]. EUS and POCS may provide a better definition of ICPN compared with other imaging modalities. In the present case, IDUS revealed a papillary tumor in the common bile duct. Therefore, clinicians should be familiar with ICPN, and make an effort to accurately diagnose it using multiple imaging techniques.

The treatment for ICPN is oncological resection; however, the selection of the optimal surgical procedure is often challenging. Simple cholecystectomy is sufficient for ICPN limited to the gallbladder mucosa without invasion. However, approximately half of the ICPN cases have an invasive component [1]. Moreover, some patients have ICPNs suspected of common bile duct invasion due to a protruding tumor from the gallbladder to the common bile duct [4, 5]. In the present case, CT, MRI, and ERCP findings indicated that the tumor was located in the distal bile duct, and IDUS suggested that the tumor had invaded the subserosa of the bile duct; therefore, we decided to perform PPPD. Moreover, intraoperative findings showed a thickened and indurated gallbladder wall, suggesting advanced gallbladder carcinoma; thus, we performed an extended cholecystectomy in addition to PPPD. Intraoperative frozen-section analysis of the cut end of the hepatic-sided bile duct confirmed no evidence of a tumor. To select the optimal surgical procedure, a comprehensive evaluation that considers preoperative imaging studies and intraoperative findings is essential. Notably, EUS, including IDUS, can be a useful tool for assessing tumor extension of ICPN.

Some studies have reported that ICPN with or without invasive carcinoma has a good prognosis, in contrast to other types of gallbladder carcinoma [1, 40, 41]. Adsay et al. reported that the 1-, 3-, and 5-year overall survival rates of non-invasive ICPN were 90%, 90%, and 78%, respectively [1]. In addition, the percentages of invasive ICPN were 69%, 60%, and 60%, respectively [1]. These overall survival rates are much better than those of other types of gallbladder carcinomas, which have an 18–30% 5-year survival rate [1, 42]. In contrast, a recent study reported that in a stage-matching analysis of gallbladder carcinoma, there was no difference between the prognosis of invasive carcinoma and other types of gallbladder carcinoma [43]. In our review, most ICPN patients had a good prognosis. We speculate that this was due to most ICPNs being resected at an early stage. However, our patient had advanced cancer originating from an ICPN with lymph node metastasis. Therefore, our patient was closely followed up with adjuvant chemotherapy.

The optimal choice of surgical procedure, including extended cholecystectomy, bile duct resection, and pancreaticoduodenectomy is essential for achieving complete oncological resection of the tumor. In addition, close postoperative follow-up is crucial for patients with ICPN, especially those with advanced cancer arising from the tumor, in accordance with other types of gallbladder carcinoma.

## 4. Conclusions

Accurate preoperative diagnosis of ICPN, including the extent of tumor invasion, is challenging; however, both EUS and POCS are effective tools for resolving these challenges. ICPN has been recognized as a tumor with a better prognosis compared with other types of gallbladder carcinoma; however, a recent study reported that the prognosis of these tumors is equivalent. The optimal choice of surgical procedure and close postoperative follow-up are essential for patients with ICPN, especially those with advanced cancer arising from the tumor.

## Figures and Tables

**Table 1:** Overview of the recent literature on ICPN.

Case	First author	Age (year)	Gender	Symptom	Imaging findings	Preoperative diagnosis	Surgical procedure	Invasive component	Lymph node metastasis	Adjuvant therapy	Outcome
1	Sato [8]	77	Female	Epigastric pain	Gallbladder tumor	Not mentioned	SC	Yes	No	No	Alive 10 months
2	Dettoni [9]	62	Female	Abdominal pain	Gallbladder tumor	Gallbladder cancer	EC	Yes	No	No	Alive 29 months
3	Meguro [10]	54	Female	Epigastric pain	Papillary tumor	Gallbladder tumor	EC, extrahepatic bile duct resection, and choledochojejunostomy	Yes (complicated with mixed adenoneuroendocrine carcinoma)	No	No	Alive 24 months
4	Hashimoto [4]	58	Female	Jaundice	Papillary tumor	Gallbladder cancer	EC, PD	Yes	No	No	Recurrence 16 months after surgery
5	Michalinos [11]	28	Female	Epigastric pain	Dilation of common bile duct	Choledochal cyst	SC, extrahepatic bile duct resection, and choledochojejunostomy	No	No	No	Not mentioned
6	Sato [12]	64	Male	Epigastric pain	Cystic tumor	Not mentioned	SC	Yes	No	No	Alive 8 months
7	Páez Cumpa [13]	39	Male	Epigastric pain	Gallbladder tumor	Not mentioned	SC	No	No	No	Alive 6 months
8	Unno [14]	74	Male	No	Papillary tumor	Gallbladder cancer	EC	Yes	No	No	Alive 3 months
9	Mizobuchi [15]	74	Female	No	Papillary tumor	Gallbladder cancer	EC	No	No	No	Not mentioned
10	Mizobuchi [15]	61	Female	No	Papillary tumor	Gallbladder cancer	EC	Not mentioned	No	No	Not mentioned
11	Mizobuchi [15]	83	Male	No	Papillary tumor	Not mentioned	EC	Not mentioned	No	No	Not mentioned
12	Sakai [5]	74	Male	Jaundice	Papillary tumor	Gallbladder cancer	EC, extrahepatic bile duct resection, and choledochojejunostomy	Yes	No	No	Not mentioned
13	Muranushi [16]	70	Male	No	Gallbladder tumor	Gallbladder cancer	EC	No	No	No	Alive 42 months
14	Hara [17]	71	Male	No	Papillary tumor	ICPN	SC	No	No	No	Not mentioned
15	Fujii [6]	59	Male	Fatigue	Papillary tumor	ICPN	SSPPD after SC	Yes	No	Tegafur/gimeracil/oteracil	Alive 2 months
16	Yokode [18]	58	Female	Fever	Papillary tumor	ICPN	EC and SSPPD	Yes	No	No	Alive 6 months
17	Sarıtaş [19]	52	Female	Right upper quadrant pain	Papillary tumor	Gallbladder tumor	SC	No	No	No	Not mentioned
18	Park [20]	78	Female	Epigastric pain	Gallbladder tumor	Not mentioned	SC	Yes (complicated with angiosarcoma)	No	No	Alive 3 months
19	Fraga [21]	85	Female	Epigastric pain	Gallbladder tumor	Not mentioned	EC	Yes (complicated with NEC)	No	No	Death 3 months after surgery
20	Sciarra [22]	66	Female	Abdominal pain	Papillary tumor	Not mentioned	EC	Yes (complicated with MiNEN)	No	No	Alive 5 months
21	Oh [23]	79	Female	Abdominal pain	Papillary tumor	ICPN	SC	Yes	No	No	Alive 36 months
22	Iwasaki [24]	52	Female	No	Papillary tumor	Gallbladder cancer	EC, extrahepatic bile duct resection, and choledochojejunostomy	No	No	No	Alive 5 months
23	Oba [25]	78	Female	Jaundice and epigastric pain	Papillary tumor	Gallbladder tumor	SC	Yes	No	No	Alive 12 months
24	Logrado [26]	71	Female	Epigastric pain	Wall thickness of the gallbladder	Not mentioned	SC	No	No	No	Alive 30 months
25	Iseki [7]	83	Male	No	Papillary tumor	Distal bile duct cancer	SSPPD	Yes	N0	No	Alive 20 months
26	Kuniyoshi [27]	86	Female	Jaundice	Papillary tumor	ICPN	SC	No	No	No	Alive 12 months
27	Ismail [28]	48	Female	No	Gallbladder tumor	Gallbladder tumor	SC	No	No	No	Not mentioned
28	Aida [29]	65	Female	No	Papillary tumor	Gallbladder cancer	EC	No (complicated with xanthogranulomatous cholecystitis)	No	No	Alive 3 months
29	Dörr [30]	77	Female	No	Wall thickness of the gallbladder	Chronic cholecystitis	SC	Yes	No	No	Not mentioned
30	Shimada [31]	69	Male	No	Papillary tumor	Gallbladder cancer	EC	Yes	No	No	Alive 12 months
31	Wong [32]	49	Male	No	Gallbladder tumor	Not mentioned	SC	No	No	No	Not mentioned
32	Trisal [33]	48	Male	Epigastric pain	Wall thickness of the gallbladder	Not mentioned	SC	No (complicated with xanthogranulomatous cholecystitis)	No	No	Alive 6 months
33	Watanabe [34]	79	Female	No	Gallbladder tumor	Gallbladder cancer	EC after subtotal cholecystectomy	Yes	No	No	Alive 8 months
34	Oishi [35]	60s	Female	No	Papillary tumor	ICPN	EC and resection of the extrahepatic bile duct	No	No	No	Alive 24 months
35	Limaiem [36]	76	Male	Right upper quadrant pain	Papillary tumor	Not mentioned	EC	Yes	No	No	Not mentioned
36	Scarola [37]	80	Male	Fatigue	Gallbladder tumor	Not mentioned	EC	Yes	No	No	Alive 6 months
37	Mruthyunjayappa [38]	70s	Male	Right upper quadrant pain	Gallbladder tumor	Gallbladder cancer	EC	Yes (complicated with NEC)	No	No	Death 50 months after surgery
38	Koike [39]	44	Male	No	Gallbladder tumor	Cholesterol polyp or pyloric-type adenoma	SC	No	No	No	Not mentioned
39	Our case	75	Male	Jaundice	Papillary tumor	Distal bile duct cancer	PPPD and EC	Yes	Yes	Tegafur/gimeracil/oteracil	Alive 12 months

Summary		Age	Male	No symptom	Papillary tumor	ICPN	SC	Yes	Yes	Tegafur/gimeracil/oteracil	Follow-up duration,
		66.3	17	17 (43.6)	22 (56.4)	6 (15.4)	14 (35.9)	22	1	2	Months
		(28–86)	Female	Abdominal pain	Gallbladder tumor	Gallbladder cancer	EC	No	No	No	14.2 (2–50)
			22	16 (41.0)	12 (30.8)	13 (33.3)	21 (53.8)	17	38	37	Alive
				Jaundice	Wall thickness of the gallbladder	Gallbladder tumor	Resection of the extrahepatic bile duct				24
				5 (12.8)	3 (7.8)	4 (10.3)					Recurrence
							5 (12.8)				3
							PD				Death
							5 (12.8)				2

Data are expressed as the mean (range) or number (%).

ICPN: intracholecystic papillary neoplasm; SC: simple cholecystectomy; EC: extended cholecystectomy; PD: pancreaticoduodenectomy; SSPPD: subtotal stomach-preserving pancreaticoduodenectomy; PPPD: pylorus-preserving pancreaticoduodenectomy; NEC: neuroendocrine carcinoma; MiNEN: mixed neuroendocrine-non-neuroendocrine neoplasm.

## Data Availability

Data supporting this research article are available from the corresponding author or first author upon reasonable request.
